# Spatiotemporal structure function coupling optimizes Taihang Mountains forest grassland ecological networks for water conservation

**DOI:** 10.1016/j.isci.2026.116974

**Published:** 2026-07-30

**Authors:** Chongyang Zhao, Jiaxin Yu, Zhongze Hou, Hao Sun, Yaqin Zhang, Yuheng Wang, Zhihao Zhang, Tong Gao, Teng Niu

**Affiliations:** 1 School of Architecture & Art, Shijiazhuang Tiedao University, Shijiazhuang 050043, China; 2 Shijiazhuang Forestry Bureau, Shijiazhuang, China; 3 Research Center for Sustainable Development of Human Settlements, Shijiazhuang Tiedao University, Shijiazhuang 050043, China

**Keywords:** taihang mountain area, forest-grassland ecological space network, water conservation, complex network, topological characteristics

## Abstract

Mountain ecosystems are critical hubs for regional water security, yet they are highly vulnerable to habitat fragmentation. Effective conservation requires coupling complex spatial configurations directly with essential ecosystem services. Using the Taihang mountains (2000–2020) as a case study, key forest-grassland ecological sources were identified across the region. Spatial networks were then constructed via the Minimum Cumulative Resistance model to systematically assess their water conservation capacity. The analysis revealed strong positive correlations (r > 0.6, *p* < 0.01) between topological indicators, specifically degree and PageRank, and water conservation. This indicates that spatial structure significantly regulates regional hydrological functions. Consequently, a differentiated Keystone-Linkage optimization strategy was proposed, prioritizing the addition of 51 strategic corridors. This enhancement delayed the network’s structural collapse threshold under simulated attacks by 29.5% compared to random restoration. This framework provides a practical, transferable method for designing resilient, function-oriented ecological security patterns in complex mountain landscapes.

## Highlights

• Complex network theory and MCR models mapped forest-grassland ecological networks
• Network topological metrics like degree strongly correlate with water conservation
• The differentiated Keystone-Linkage optimization strategy maximized water services
• The targeted addition of 51 corridors delayed network structural collapse by 29.5%

## Graphical abstract

**Figure:**
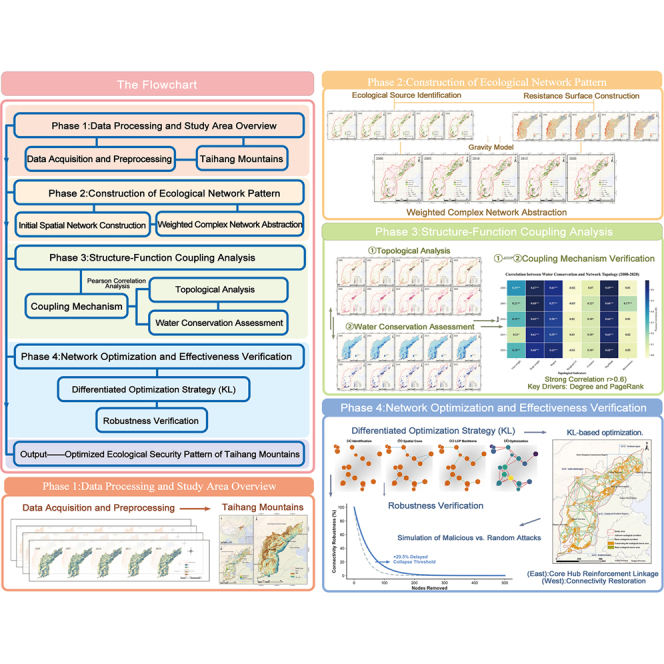

## Teaser

Environmental science; Ecology

## Introduction

Mountain ecosystems are essential for regulating hydrological cycles, maintaining soil stability, and supporting biodiversity, providing a high level of ecosystem services that benefit vast downstream regions.^1^ Specifically, the Taihang Mountains represent a region of immense strategic value, serving as a critical ecological shield and primary “water tower” for northern China. However, these landscapes are exceptionally sensitive to human activities. Increasing pressures from deforestation, mining, and urbanization have led to widespread landscape fragmentation, which disrupts the natural flow of energy and materials.^2^ This fragmentation not only threatens species survival but also critically weakens the ability of mountains to provide vital regulating services such as water conservation, thereby posing a direct risk to regional water security.^3^^,^^4^ In this context, designing well-connected and functional ecological networks has emerged as a major priority for achieving regional sustainability and resilience.^5^^,^^6^

The “patch-corridor-matrix” model serves as the foundational framework for landscape planning.^7^^,^^8^ Within this system, core habitats—such as intact forests—act as ecological sources that generate functional flows.^9^ However, these flows face resistance from the surrounding land-use matrix. Consequently, efficient corridors are essential to ensure the continuity of critical processes, such as water and nutrient cycling.^10^ To quantify these patterns, the application of graph theory has evolved significantly. Early studies primarily employed binary indices, such as the Integral Index of Connectivity (IIC) and probability of connectivity (PC), to evaluate habitat availability at the patch level.^11^ Subsequently, the focus shifted toward complex network analysis. This approach incorporates multidimensional indicators—specifically degree centrality, betweenness centrality, and network robustness—to accurately characterize how network configurations facilitate ecological processes.^12^^,^^13^ Most recently, researchers have integrated multi-scale topological attributes to better capture the spatial heterogeneity of ecological functions.^14^ In mountainous terrains, these metrics serve as intrinsic indicators of hydrological capacity. High-centrality patches act as “hydrological sponges” for rainfall interception, while structural corridors function as physical “buffer zones” that modulate runoff and prolong infiltration.^15^

Despite the widespread application of ecological network models,^16^ two critical limitations hinder their practical effectiveness in the Taihang Mountains. First, the quantitative response of water conservation services to network topological evolution remains underexplored. Most existing studies rely on the theoretical assumption that “enhanced connectivity equals improved function,” yet they often lack direct statistical evidence linking specific topological metrics (e.g., degree centrality) to actual water retention capacity over long temporal scales (2000–2020). Without this statistical validation, conservation planning risks being guided by abstract concepts rather than proven functional drivers. Second, optimization strategies often lack spatial differentiation. Conventional approaches typically employ a “one-size-fits-all” method—simply adding corridors between the closest patches—while neglecting the hierarchical importance of nodes. This uniform strategy fails to identify and prioritize “Keystone Nodes” that are critical for hydrological regulation in complex terrains, leading to inefficient resource allocation.^17^ This necessitates a differentiated strategy that adapts conservation actions to local constraints.

To address these gaps, this study introduces a “topology-function” coupling framework. Rather than relying solely on theoretical connectivity models, we integrate statistical analysis to quantify the spatial association between network topological metrics and water conservation assessment results. This approach allows us to identify which specific structural configurations correspond to higher functional performance. Based on this quantitative evidence, we develop a differentiated Keystone-Linkage (KL) optimization strategy that specifically reinforces structures critical for hydrological regulation, tailored to the complex terrain of the Taihang Mountains. Specifically, this study aims to: (1) map the spatiotemporal evolution of the ecological network from 2000 to 2020; (2) examine the correlation between topological characteristics and water conservation capacity; and (3) propose and validate an optimization plan that simultaneously enhances structural robustness and functional potential.

## Results

### Study area

**Figure 1:**
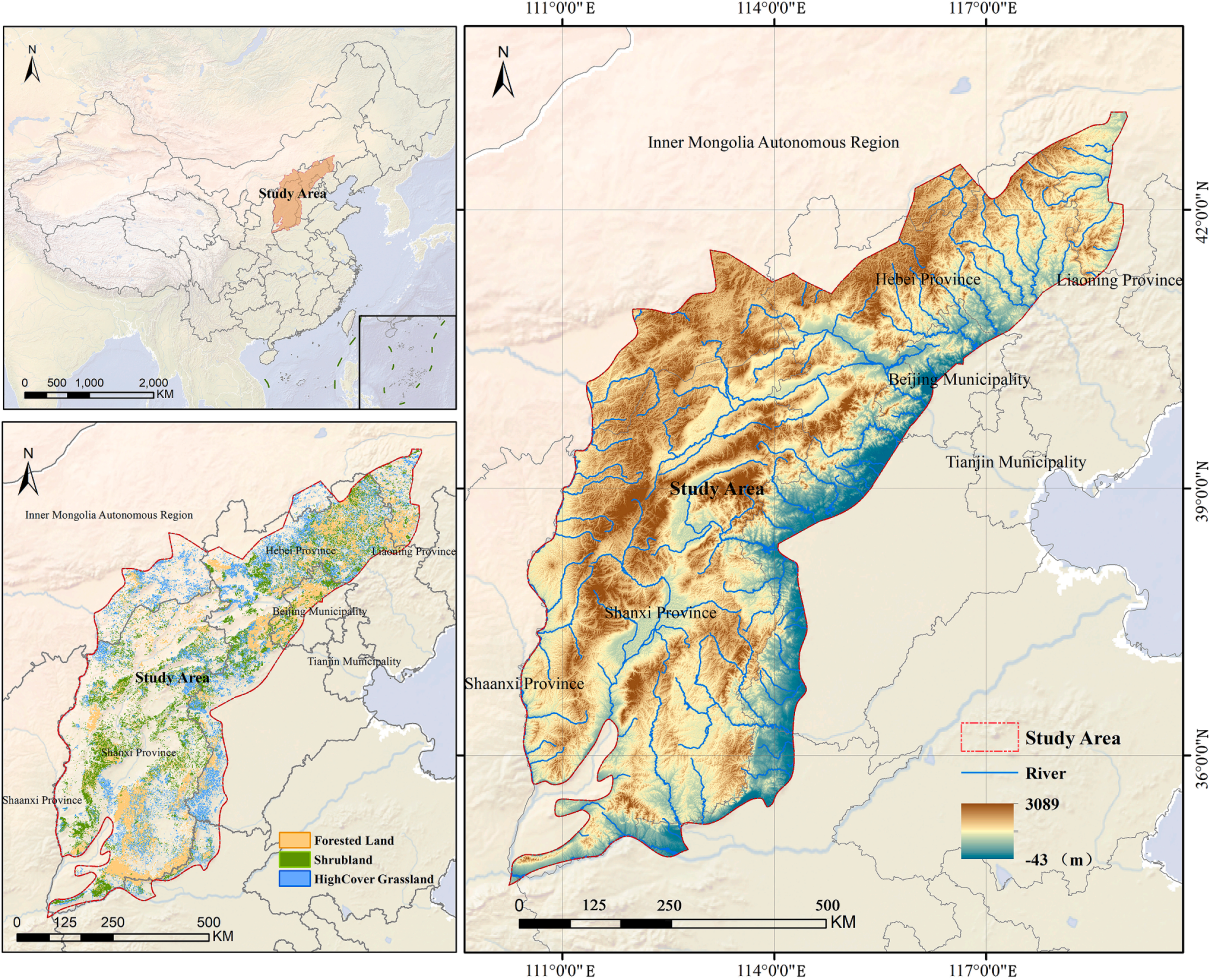
Geographical location of research area

The Taihang Mountains, extending across northern China (36°–42°N, 110°–116°E), serve as a pivotal geographical boundary distinguishing the Loess Plateau from the North China Plain (Figure 1).^18^ This region is strategically designated as a critical ecological barrier for the Beijing-Tianjin-Hebei urban agglomeration, playing an irreplaceable role in regional ecological security.^19^ Characterized by a warm temperate continental monsoon climate, the area experiences an average annual precipitation of approximately 500–600 mm, with nearly 70% concentrated in the summer months, creating distinct seasonal hydrological dynamics.^20^ The terrain is complex and rugged, with elevations ranging from 200 m to over 2,800 m, which fosters a rich mosaic of vegetation types. The dominant vegetation includes temperate deciduous broad-leaved forests, shrublands, and montane meadows, which collectively function as a “green reservoir” regulating surface runoff and soil erosion.^21^ As the primary recharge zone for the Haihe River Basin, the Taihang Mountains are essential for sustaining the water supply of downstream cities. However, the ecosystem remains fragile; long-term anthropogenic disturbances and landscape fragmentation have intensified the conflict between ecological protection and resource development, making the optimization of its ecological network urgent.^22^^,^^23^

### Data sources and processing methods

This study utilized multi-source geospatial data for the years 2000, 2005, 2010, 2015, and 2020. The primary datasets, including land use/cover, digital elevation model (DEM), meteorological data, and normalized difference vegetation index (NDVI), were acquired from various official data repositories. To ensure spatial consistency for all subsequent analyses, all raster datasets were resampled to a uniform spatial resolution of 200 m × 200 m using the nearest neighbor method in ArcGIS. A detailed summary of the data sources and their processing is provided in Table 1.

**Table 1:** Summary of data sources and processing

Data type	Source/provider	Original resolution/scale	Year (s)	Processing method and primary use
Land use/cover (LULC)	resource and environmental science and data center (RESDC)	1000 m raster	2000–2020	resampled to 200 m. Used for identifying ecological sources and as a primary factor in resistance surface calculation.
Digital elevation model (DEM)	geospatial data cloud (SRTM)	90 m raster	N/A	resampled to 200 m. Used to derive the slope factor for the resistance surface.
Meteorological data (precipitation, temperature)	cold and arid regions scientific data center (CARS), CAS	station-based daily data	2000–2020	interpolated into 200 m raster surfaces. Used for calculating evapotranspiration and water balance.
Normalized difference vegetation index (NDVI)	MODIS (MOD13Q1 Product)	250 m raster	2000–2020	resampled to 200 m. Used as a proxy for vegetation cover in the resistance surface calculation.
Soil type data	Chinese academy of sciences (CAS)	1:1,000,000 vector map	N/A	converted to a 200 m raster. Used to derive soil organic matter and soil erodibility factors for resistance.
Road network	National Geomatics Center of China	vector data	2000–2020	used for line density analysis to calculate road density as a resistance factor.
Water system	Ministry of Water Resources of China	vector data	2000–2020	used for line density analysis to calculate water network density as a resistance factor.

### Research framework

**Figure 2:**
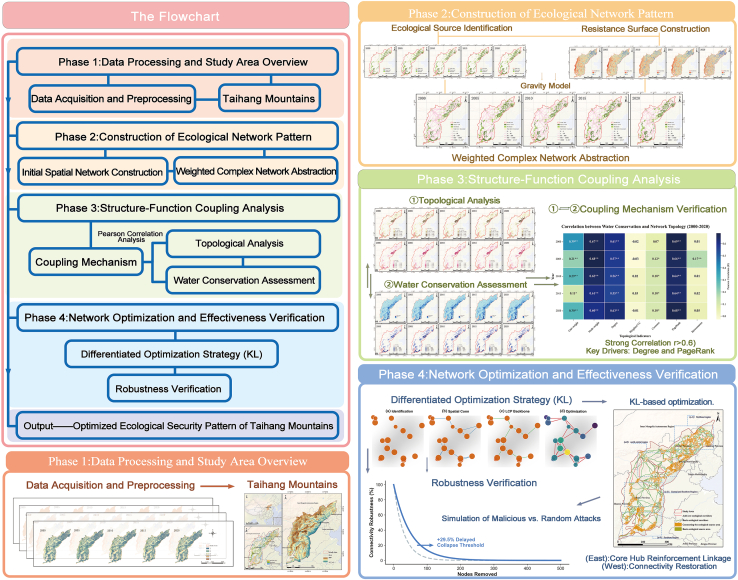
The methodological framework of this study

This study establishes a systematic framework structured around four key stages: identification, construction, coupling, and optimization (Figure 2). Initially, valid forest-grassland sources are delineated via a multi-factor evaluation system (based on the Entropy Weight Method) and interconnected across a Minimum Cumulative Resistance (MCR) surface. To capture the intensity of ecological interactions, the initial binary graph is transformed into a weighted complex network using the Gravity Model. By integrating topological metrics (e.g., PageRank, Degree) with the Water Balance Equation, we simultaneously characterize network structure and quantify water conservation services, employing Pearson’s correlation analysis to unveil the intrinsic “structure-function” coupling mechanism. Guided by these quantitative insights, a differentiated “Keystone-Linkage (KL)” strategy is proposed to reinforce core hubs and bridge peripheral gaps, with its effectiveness validated through robustness assessments under simulated attack scenarios.

### Spatiotemporal dynamics of the ecological network

#### Spatiotemporal patterns of ecological sources

**Figure 3:**
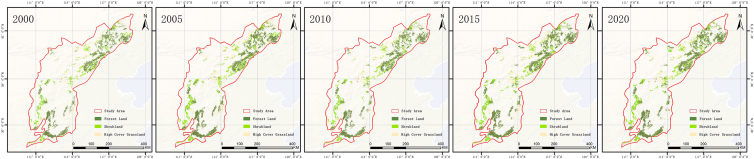
Spatiotemporal evolution of forest-grassland ecological sources in the Taihang Mountains from 2000 to 2020

The spatiotemporal evolution of ecological sources reveals a fluctuating trajectory of “sharp decline followed by partial recovery,” closely mirroring the region’s land-use policies. Temporally, the forest-grassland ecological space underwent significant stress and subsequent restoration (Figure 3). Between 2000 and 2005, intensive mining activities and rapid urbanization in the foothills caused a severe 43% contraction in high-quality forest and grassland patches. However, this degradation was effectively reversed post-2005 following the implementation of national ecological projects (e.g., the Taihang Mountain Greening Project). By 2020, the number of ecological sources rebounded to 452, with a notable re-expansion of shrublands and high-coverage grasslands in the restoration zones, physically reinforcing the network’s foundation.

Spatially, despite the quantitative recovery, the landscape maintains a persistent “Dense East - Sparse West” imbalance. Our analysis identifies that high-quality sources are structurally clustered in the western Beijing conservation area (northeast) and the east-central foothills, forming a continuous forest-grassland belt. In contrast, the high-altitude western region remains fragmented, characterized by scattered, small-scale grassland patches that fail to form a cohesive barrier. This uneven distribution highlights a critical structural vulnerability: While the total area of the forest-grassland space has been restored, its spatial configuration remains polarized, leaving the western ecological security barrier functionally weak.

#### Ecological resistance surface

**Figure 4:**
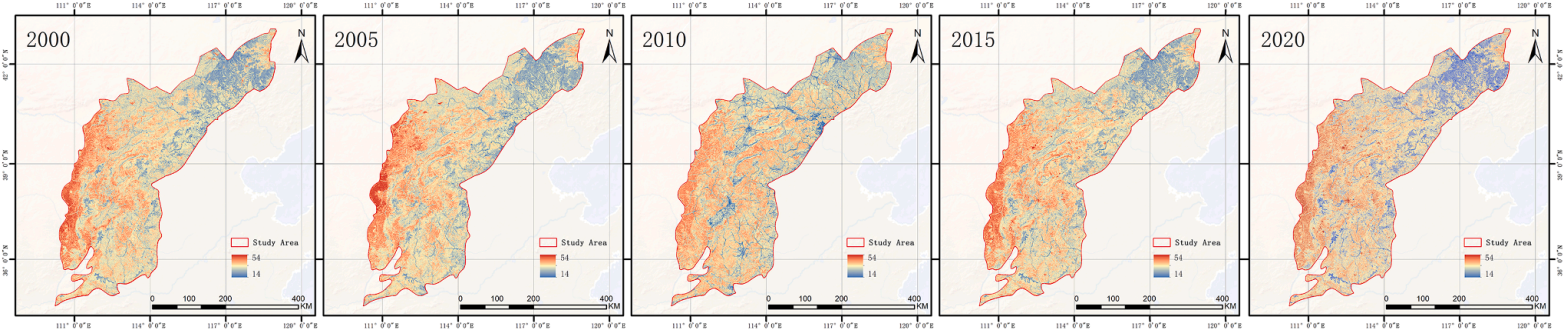
Ecological resistance surface

The ecological resistance surface, which represents the difficulty of ecological flow, displayed a clear and stable spatial gradient across the Taihang Mountains (Figure 4). Resistance values were consistently high in the northwest, a region characterized by arid conditions and complex topography. In contrast, the forested southeastern part of the range exhibited the lowest resistance. While this overall pattern remained stable, we observed a localized increase in resistance around major cities such as Handan and Xingtai after 2015, highlighting the growing impact of urban expansion on landscape connectivity.

#### Structure and characteristics of ecological corridors

**Figure 5:**
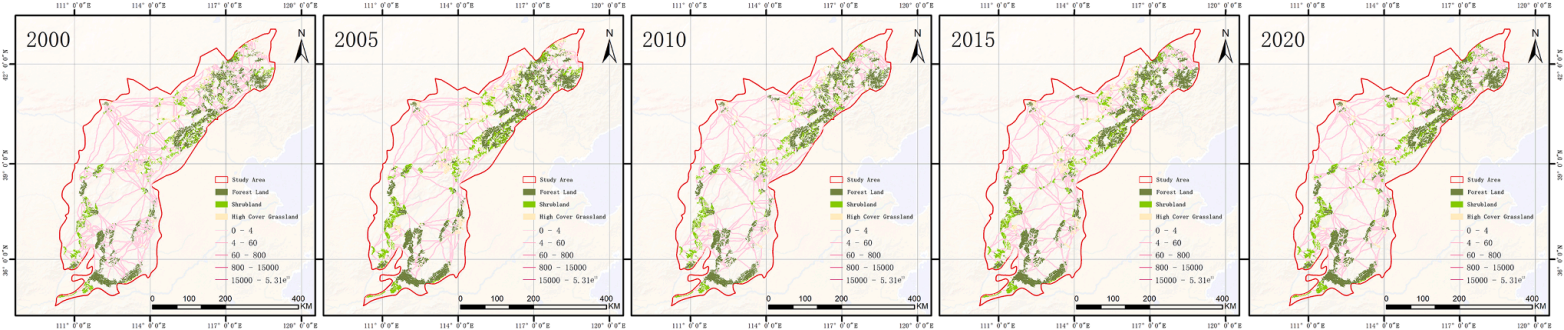
Spatiotemporal evolution of ecological corridor distribution and interaction intensity (edge weight) in the Taihang Mountains (2000–2020)

The evolution of ecological corridors reveals a critical structural imbalance where numerical recovery has not translated into effective regional connectivity. The network connectivity fluctuated significantly between 2000 and 2020. Intensive land use initially caused a sharp 38% decline in corridor numbers by 2005, but subsequent large-scale afforestation programs successfully restored the total count to 983. However, this recovery primarily consisted of short-distance links within local restoration zones rather than the long-range connections required for regional ecological stability. Spatially, the network exhibits a polarized distribution marked by a dense eastern section and a sparse western interior (Figure 5). The east-central region benefits from continuous forest cover to form a functional artery that facilitates efficient ecological flows and supports high water conservation capacity. In contrast, the high-altitude western region remains a connectivity blind spot despite its role as a critical headwater area. The sparse and fragmented corridors here create a fracture zone that blocks the spillover of ecosystem services from core areas to the periphery.

Consequently, future network optimization must transcend simple “densification.” Instead, it requires a differentiated strategy: reinforcing the efficiency of the eastern artery while prioritizing “strategic bridging” across western high-resistance barriers. By identifying low-cost pathways to reconnect these fractured headwaters, the network can shift from a fragmented state to a cohesive system, ensuring the effective spillover of water conservation services.

#### Topological characteristics: network connectivity and functional heterogeneity

This section applies complex network theory to decode the “structure-function” coupling mechanism of the Taihang Mountains ecosystem. By transforming the spatial landscape into a topological graph, we analyze how the network’s configuration governs the efficiency of regional ecological flows. Specifically, we examine the evolution of overall connectivity, identify the hierarchical “key nodes” that sustain stability, and reveal the spatial mismatch between ecological potential and actual structural support. This multi-dimensional analysis provides the theoretical basis for the subsequent optimization strategies.

#### Evolution of network connectivity

**Figure 6:**
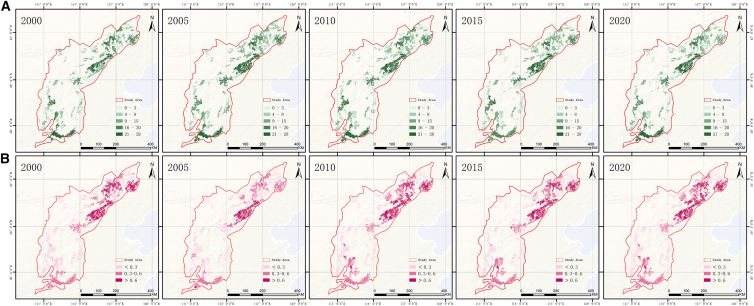
Spatiotemporal evolution of topological connectivity indicators in the ecological network (2000–2020) (A) Degree of ecological sources: represents the number of direct connections for each node, highlighting the “hub” status of the Beijing-Hebei transition zone. (B) Weighted clustering coefficient: reflects the degree of local node clustering. The steady increase indicates that fragmented patches are gradually forming cohesive groups.

The ecological network has gradually evolved into a stable structure with high local clustering, driven largely by ecological restoration projects. The Weighted Clustering Coefficient increased from 0.43 in 2000 to 0.57 in 2020, as shown in Figure 6. This growth was particularly rapid between 2005 and 2010, indicating that the Taihang Mountain Greening Project effectively reconnected fragmented forest patches into cohesive groups. Spatially, the network relies on a strong center in the Beijing-Hebei transition zone. Areas such as Fangshan and Laiyuan consistently show the highest Degree values, peaking at 28. This region acts as a central hub that facilitates frequent material and energy exchange between the North China Plain and the Shanxi Plateau, directly supporting the stability of regional water cycles.

#### Identification of key nodes

**Figure 7:**
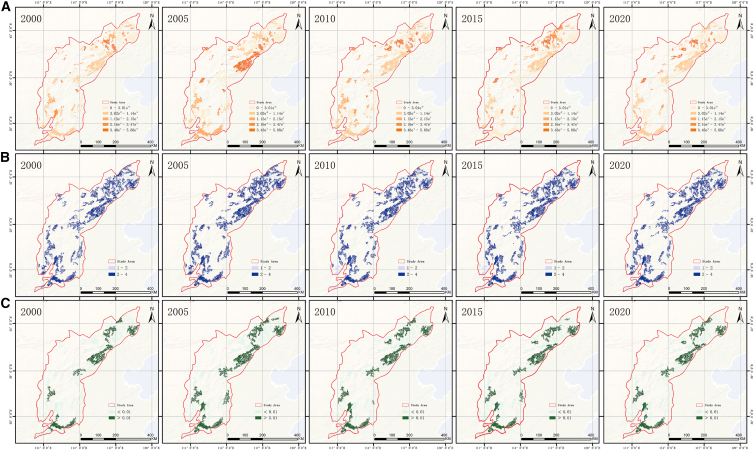
Spatial identification of key nodes based on centrality metrics (2020) (A) Betweenness centrality: identifies “stepping stones” that bridge different regions, crucial for maintaining long-distance connectivity. (B) Coreness: distinguishes the network’s core backbone (high values) from peripheral nodes (low values). (C) PageRank value: ranks the global influence of nodes, pinpointing the most critical “ecological anchors” in the northern region.

The structural integrity of the network depends on a few specific key nodes rather than being evenly distributed. Our analysis of centrality metrics in Figure 7 reveals that high-value nodes are concentrated in the central and northern regions, such as Wuling Mountain and Xiaowutai Mountain. These patches serve as critical stepping stones that bridge isolated habitats and ensure ecological flows can cross rugged terrain. Specifically, the PageRank analysis identifies about 15 core sites that exert a major influence on the entire network’s efficiency. In contrast, the Fen River Valley consistently shows low centrality values. This indicates that rapid urbanization has weakened the connections in these marginal areas, creating functional gaps that could threaten the network’s overall stability.

#### Spatial pattern of ecological potential

**Figure 8:**
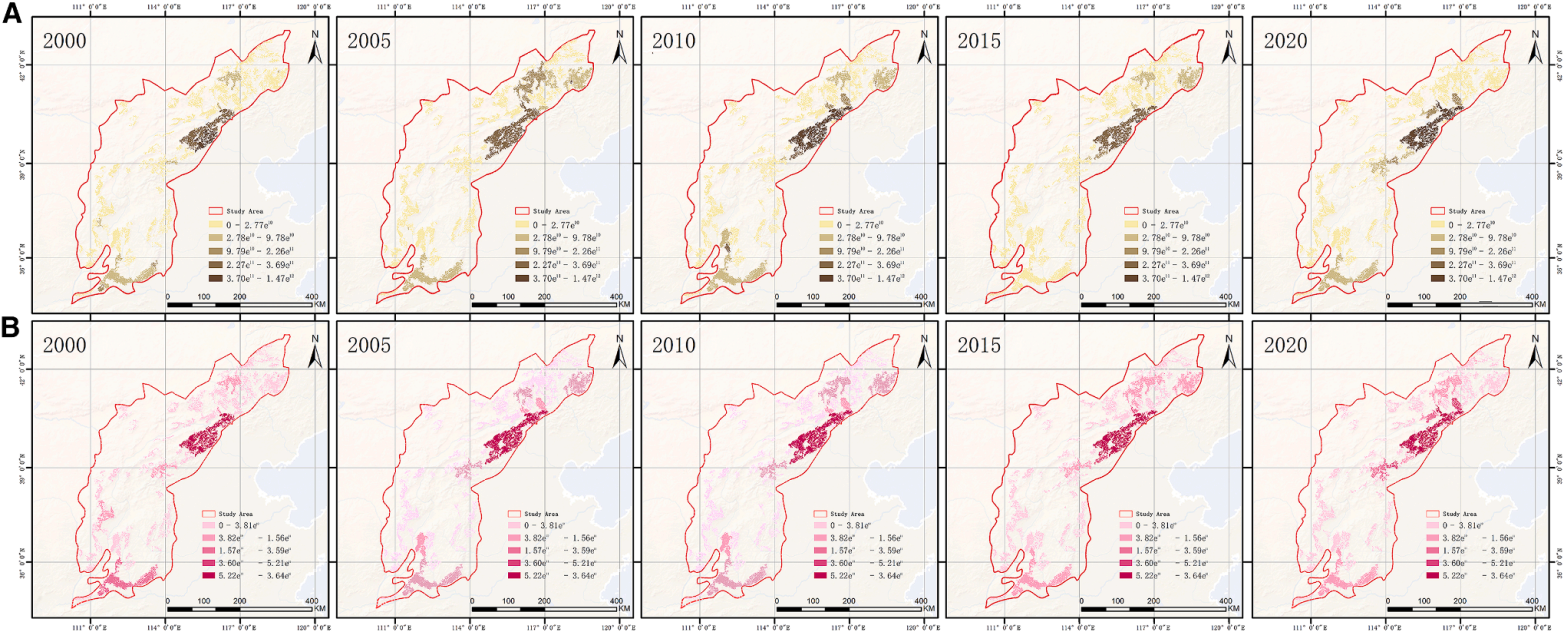
Spatial patterns of ecological functional potential and structural mismatch (2020) (A) Unit weight: represents the inherent ecological quality (e.g., habitat suitability) of each source. (B) Weight difference: represents the gap between quality and connectivity. Red areas indicate “isolated reservoirs”—high-quality sources with poor connectivity, serving as key targets for optimization.

The distribution of ecological potential shows a mismatch between topographic conditions and actual functional output. The Unit Weight metric in Figure 8 reflects the quality of ecological services in different areas. The highest values appear in the transition zones near Shijiazhuang and Handan, where sufficient rainfall and rich biodiversity combine to maximize water retention. However, a clear “East-High, West-Low” imbalance remains. While high-quality sources in western Beijing benefit from protective policies, the Wutai Mountains in the west show lower realized weights despite their complex topography. This suggests that improving the quality of these western sources is just as important as increasing their connectivity to enhance the region’s total water conservation capacity.

### Water conservation services

#### Spatial and temporal evolution of water conservation ecosystem services

**Figure 9:**
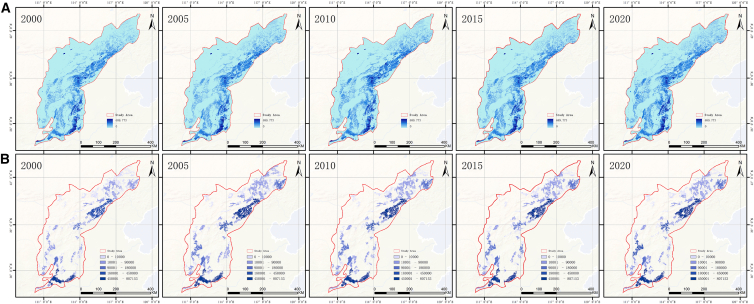
Spatiotemporal patterns of water conservation services in the Taihang Mountains (2000–2020) (A) Regional water conservation intensity (m^3^/hm^2^): displays the full-scale spatial gradient, highlighting the “High East–Low West” distribution across the entire landscape. (B) Water conservation volume in ecological sources (m^3^): quantifies the specific functional contribution of individual network nodes, identifying the core reservoirs and vulnerable patches.

From 2000 to 2020, the water conservation function in the Taihang Mountainous Region exhibited a distinct “High in the East, Low in the West” spatial gradient (Figure 9A).

The eastern and northeastern regions, including the Beijing Xishan, Hebei Wuling Mountains, and the piedmont transition zones, emerged as high-value water conservation areas. This is primarily due to the continuous woodland coverage (exceeding 60%) and the interceptive effect of the eastern slopes on monsoon rainfall. The average water conservation value in these areas remained above 4,000 *m*^3^/*hm*^2^, with a local peak reaching 6,897.73 *m*^3^/*hm*^2^ in 2010. In contrast, the western regions, particularly the Lvliang mountains and the areas adjacent to the Loess Plateau, showed significantly weaker water conservation capacity. In these western zones, values consistently remained later in discussion 2,000 *m*^3^/*hm*^2^ due to fragmented topography, sparse vegetation cover (<30%), and the cumulative impact of mining and farming activities.

The water conservation volume of specific ecological sources further highlights the functional dominance of large forest patches, ranging from 0 to 807,153 *m*^3^ (Figure 9B).

High-value sources (volume >450,001 *m*^3^) are concentrated in the eastern forested ridges, such as the Xiaowutai Mountains. These areas, characterized by stable NDVI values (>0.7), form the core barrier for regional water security. While the total number of medium-value sources (180,001–450,000 *m*^3^) increased by 12% after 2010 thanks to the “Greening Project,” the western landscape remains dominated by low-value patches (volume <90,000 *m*^3^). These western patches suffer from poor ecosystem stability and high soil erosion, identifying them as the primary targets for the subsequent network optimization.

#### Coupling analysis between water conservation services and network structural characteristics

**Figure 10:**
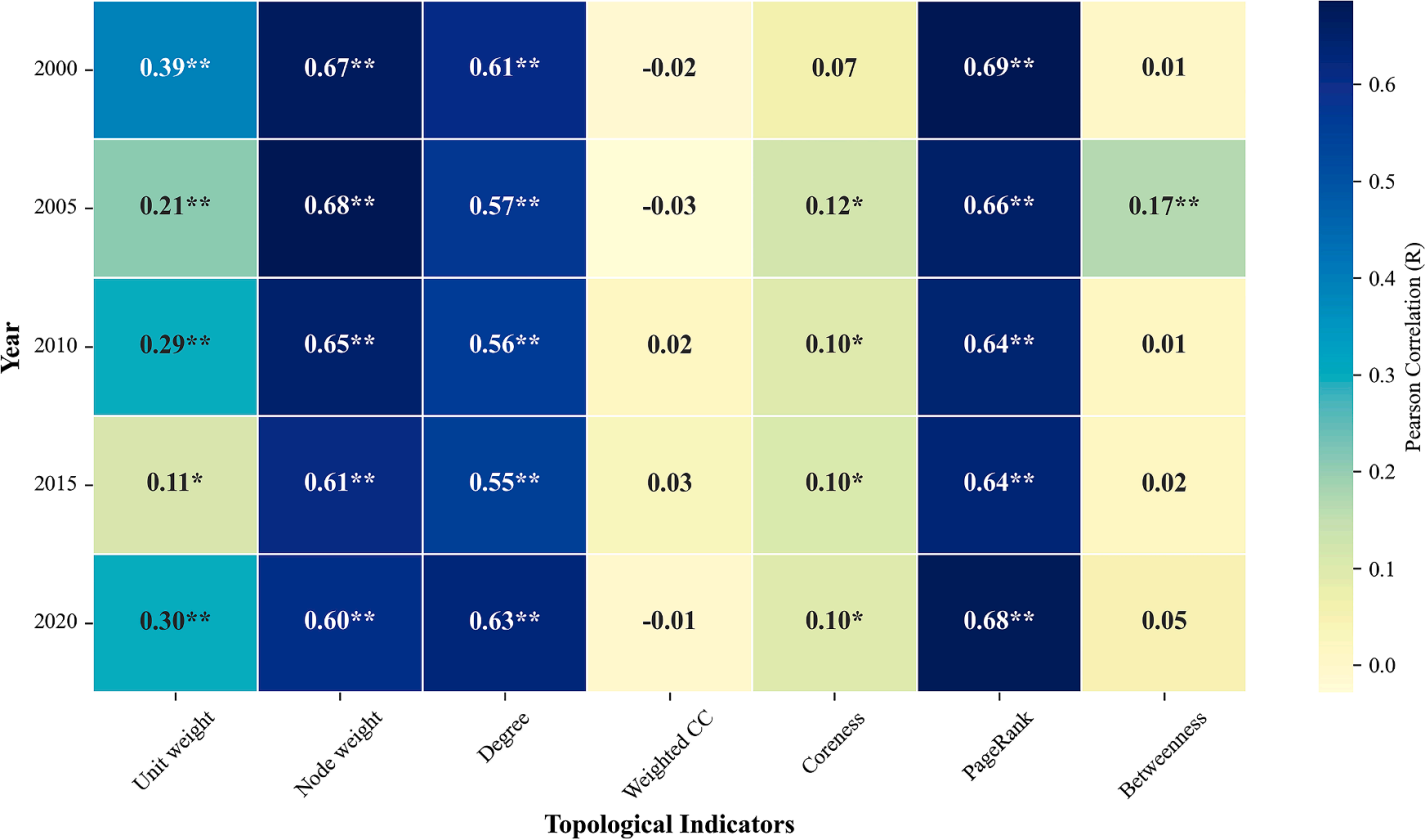
Correlation between water conservation services and topological characteristics

Statistical coupling analysis confirms that the network’s topological configuration is a primary driver of its water conservation efficacy (Table 2; Figure 10).A highly significant positive correlation (*p* < 0.01) exists between water conservation depth and key connectivity metrics, particularly Degree (*R* > 0.65) and PageRank (*R* > 0.6). This suggests that sources with more corridor connections and greater global influence are more efficient at facilitating ecological flows, thereby enhancing their hydrological regulation capacity. Similarly, Node Weight shows a strong correlation (*R* > 0.6), indicating that the magnitude of ecosystem energy transfer directly dictates the volume of water retained.

**Table 2:** Correlation of water-holding services with topological features

Topology	Unit weight	Node weight	Degree	Weighted clustering coefficient	Coreness	PageRank	Betweenness centrality
2000	0.39∗∗	0.67∗∗	0.61∗∗	−0.02	0.07	0.69∗∗	0.01
2005	0.21∗∗	0.68∗∗	0.57∗∗	−0.03	0.12∗	0.66∗∗	0.17∗∗
2010	0.29∗∗	0.65∗∗	0.56∗∗	0.02	0.10∗	0.64∗∗	0.01
2015	0.11∗	0.61∗∗	0.55∗∗	0.03	0.10∗	0.64∗∗	0.02
2020	0.30∗∗	0.60∗∗	0.63∗∗	−0.01	0.01∗	0.68∗∗	0.05

∗∗ indicates *p* < 0.01, which is a highly significant correlation; ∗ indicates *p* < 0.05, which is a significant correlation; blank indicates no significant correlation.

However, local clustering and coreness exhibit a much weaker influence on functional output. The Weighted Clustering Coefficient showed no significant correlation throughout the study period, implying that simple local aggregation of patches does not necessarily lead to better water conservation. Furthermore, the correlation with Coreness remained low, suggesting that a node’s proximity to the geographic center is less important than its active connectivity (Degree). These findings emphasize that to improve water security, optimization should focus on enhancing the flow and connectivity of the network rather than merely expanding the area of isolated core zones.

#### Network optimization using the Keystone-Linkage strategy

Based on the results of the Pearson’s correlation analysis, the water conservation services of the forest-grassland ecological network in the Taihang Mountains were found to be significantly correlated with the unit weights, node weights, degrees, and PageRank values of the ecological spatial network (with correlation coefficients ranging from 0.11 to 0.69 and *p* values of less than 0.05 or 0.01). The correlation between node weights and PageRank values was particularly strong (with multi-year means of 0.64 and 0.66, respectively). This highlights the significant impact of energy transfer capacity, connectivity breadth, and network importance of ecological sources on water conservation, establishing them as pivotal drivers for the enhancement of ecosystem services.

**Figure 11:**
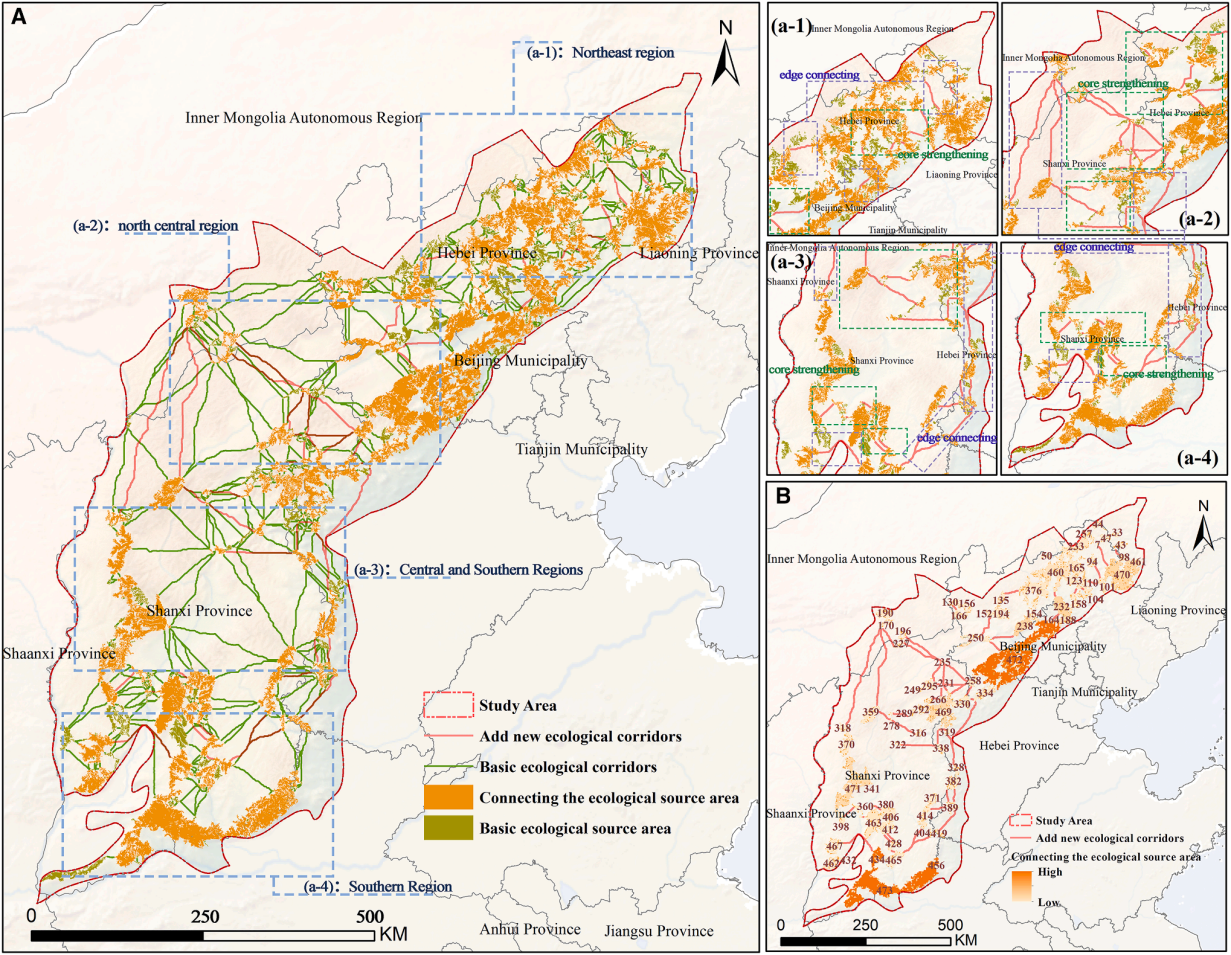
Multi-scale spatial implementation of the Keystone-Linkage (KL) optimization strategy in the Taihang Mountains (A) Comprehensive spatial layout of the optimized forest-grassland ecological network; (a1–a4) Regionalized implementation based on the “Core Strengthening-Edge Connectings” strategy. (B) Targeted extraction of newly identified corridors and topological hierarchy of associated sources.

To optimize the ecological spatial network, this study constructed a high-priority ecological source screening system using 2020 as the baseline and incorporating multiple indicators. The process involves the following steps. First, the unit weight, node weight, degree, and PageRank value are normalized to eliminate scale differences between indicators. Secondly, a weight allocation scheme is determined based on the strength of each indicator’s association with the water conservation function (12% for unit weight, 30% for node weight, 27% for degree, and 31% for PageRank value). Third, the integrated attribute value of ecological source areas is calculated using a weighted sum formula. Finally, based on the 2020 benchmark, the screening system for high-priority ecological source areas is constructed. The top 20% of ecological sources, as identified by the weighted sum formula, are selected as the optimization core due to their strong ecological energy and high connectivity, which makes them key drivers of enhanced water conservation. The spatial differentiation of the ecological resistance surface in the study area follows a “high in the northwest and low in the southeast” pattern. This is due to high resistance in the northwestern Lvliang Mountain area (due to its steep terrain) and low resistance in the southeastern Taihang Mountain area (due to its gentler terrain). Taking into account the direction of the mountain ranges and river valleys, 51 ecological corridors were added (Figure 11), forming a “Core Strengthening - Edge Connecting” pattern that is key to optimizing the water conservation function.

#### Core-area synergy optimization through high-weight corridor addition

The core aggregation area primarily encompasses the dense ecological source zones in the east-central part of the country, including Xishan Mountain in Beijing and Zhangshiyan in Hebei Province. These areas feature a large number of concentrated ecological sources with strong connectivity within the original network. The optimization strategy focuses on “strengthening the synergy of high-value areas”. Based on the gravity model, new high-weight corridors are added to enhance the efficiency with which ecological flows interact. For example, in the new connection between sources 472 and 232, both sources are ranked in the top 10% for comprehensive attribute values. Before optimization, the total weight of source 472 was 2.67 × 10^13^, and that of source 232 was 1.77 × 10^12^. The edge weight of the new corridor is 1.64 × 10^10^, which increases the total weight of the two sources by 0.06% and 1.05%, respectively. While this numerical increase is modest, the “multi-path transmission” capacity of ecological flows in the core area was significantly enhanced, thereby strengthening the spatial synergy of the water conservation function. This optimization strengthens the connection between core nodes in forest and scrub ecosystems, creating a “1 + 1>2” effect and enhancing water conservation services.

#### Low-resistance connectivity optimization for fragmented peripheral zones

The marginal fragmented area primarily comprises ecological sources such as the Luliang Mountainous Area in the northwest, where weak ecological network connectivity is the result of terrain barriers and interference from human activity. The optimization strategy focuses on “breaking spatial barriers”, using the cost path model to identify low-resistance corridors such as river valleys and gentle slopes, as well as adding new cross-regional corridors. For instance, connecting sources 399 and 463 along the Fen River valley increased the total weight of source 399 from 6.31 × 10^8^ before optimization to 6.11 × 10^7^—a 90.64% increase. This new ecological corridor establishes transmission paths for ecological flow between the western fringe and the central core area. The addition of new corridors connecting sources 250, 152, 206, and 238 to the ecological corridor of source 250 resulted in a 4.10% increase in total weight. It has become an “intermediary node,” connecting the central and eastern parts with the west and improving the water conservation capacity of the surrounding fragmented sources. This optimization process gradually integrates the grassland and shrubland ecosystems in the fringe areas into the overall network by reducing resistance to the transmission of ecological flows, thus compensating for the “gap” in regional service functions.

#### Robustness

This study assesses the impact of edge-increasing optimization on the stability of the forest-grassland ecological spatial network in the Taihang Mountains, comparing connectivity and recovery robustness before and after optimization. The analysis simulates both malicious attacks, which involve preferentially removing high-importance nodes, and random attacks, which involve randomly removing nodes, to reveal the ecological network’s ability to resist disturbances over time.

#### Connectivity robustness analysis

**Figure 12:**
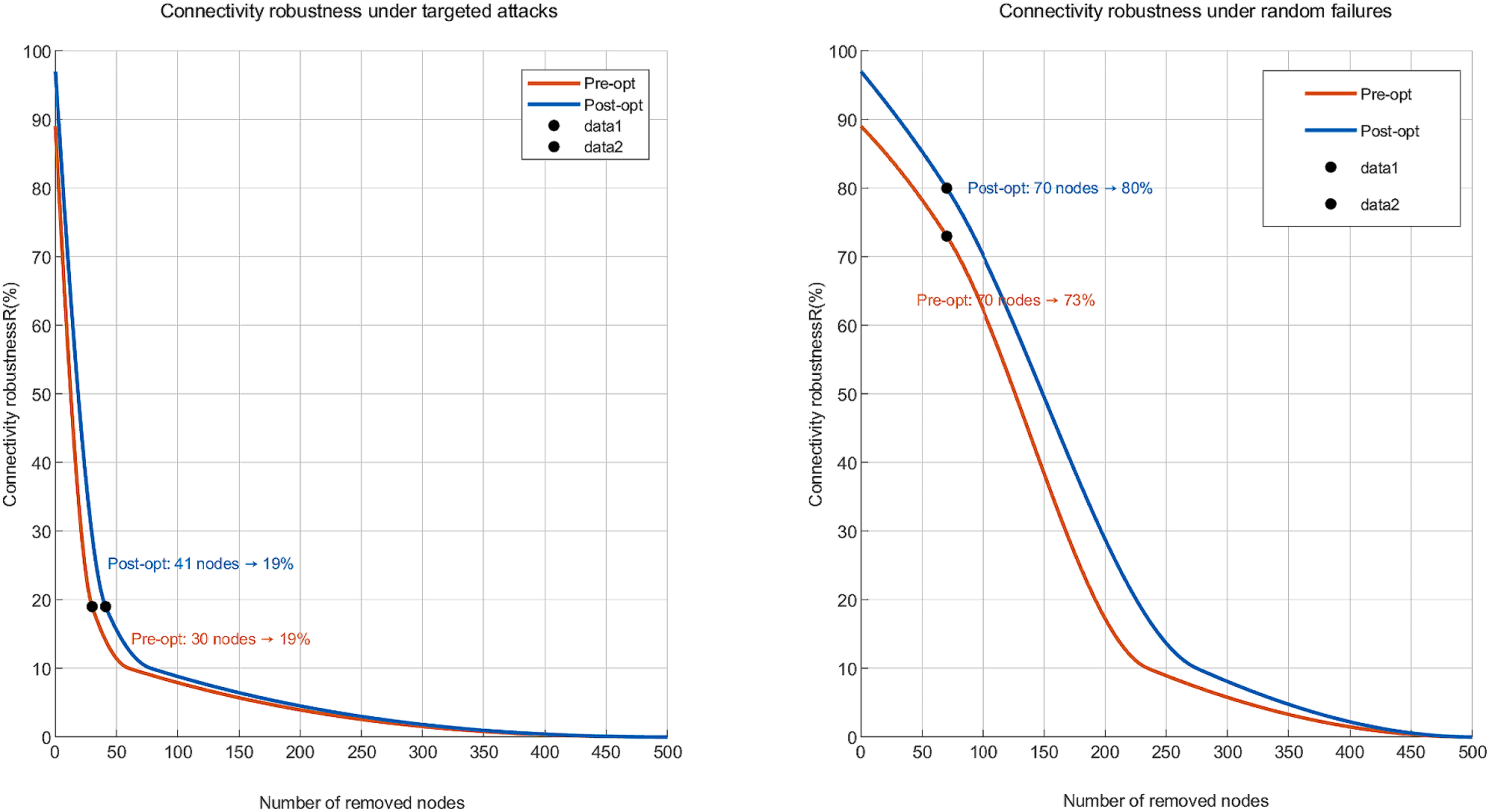
Comparative analysis of connectivity robustness in the ecological spatial network before and after optimization (2020)

The optimization significantly enhances the network’s ability to stay connected under pressure. Initially, the network was highly sensitive to the loss of core nodes. As shown in Figure 12, removing only 60 key nodes caused the connectivity robustness to drop below 0.10, leading to a near-total collapse. This shows that the original network relied too heavily on a few major hubs. After adding the new corridors, the initial robustness improved to 0.97, and the network could withstand the loss of 78 nodes before collapsing—an improvement of 30% in structural endurance. For random interference, the collapse threshold was delayed by 40 nodes, proving that the optimized structure is much more stable and no longer fails easily when a few parts are damaged.

#### Recovery robustness analysis

**Figure 13:**
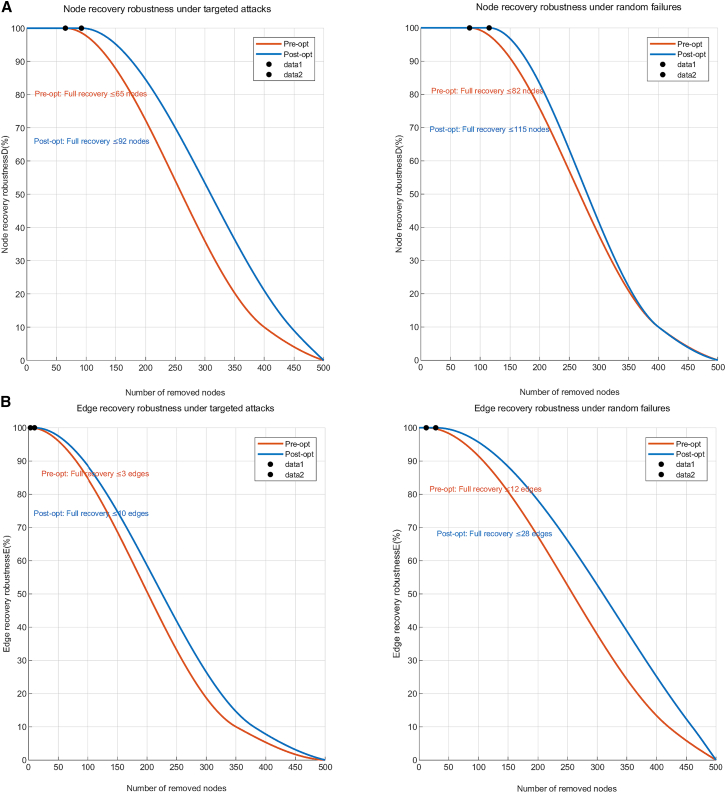
Comparative analysis of recovery robustness in the ecological spatial network before and after optimization (2020) (A) Node recovery robustness: illustrates the network’s ability to restore connectivity after node loss under malicious and random attacks. (B) Edge recovery robustness: illustrates the network’s ability to restore connectivity after corridor loss under malicious and random attacks.

The new corridors act as “backup routes,” greatly increasing the chances of the network fixing itself after damage (Figure 13). Regarding node recovery, the network can now fully recover even if 92 nodes are lost, compared to only 65 before optimization. The improvement is even more visible in corridor recovery. Under random attacks, the network’s ability to fully restore its connections increased by 133%. These additional corridors provide alternative paths for ecological flows. If one path is blocked by human activity or natural disasters, the system can use these “backup routes” to maintain its water conservation functions.

In conclusion, the edge-increasing optimization transforms the Taihang Mountainous ecological network into a more resilient system. By reducing the over-reliance on a few core hubs and providing redundant pathways, the optimized network ensures that water conservation services remain stable even under environmental stress. This structural reinforcement provides a solid foundation for long-term regional water security.^24^

## Discussion

This study focuses on the Taihang Mountainous Region, analyzing the interaction between the forest-grassland ecological spatial network structure and the region’s water conservation function between 2000 and 2020. The study systematically identifies ecological source sites, constructs the network, analyses functional correlations, and verifies optimization strategies, leading to targeted conclusions. Considering the intrinsic ecological characteristics and network structure of the study area enables us to discuss the dynamic evolution of ecological networks, topological features, functional coupling, and the practical effectiveness of optimization strategies in depth.^25^

### Structural-functional coupling: how network topology regulates water conservation

Our analysis reveals that network topology is a key driver of water conservation services. The strong positive correlation between topological metrics (such as Degree and PageRank) and water conservation depth suggests that nodes with better connectivity usually provide higher ecological functions.^26^ This is not a coincidence, but a reflection of how landscape structure supports ecosystem stability. Specifically, high-degree nodes, such as those in the Wuling Mountains, typically correspond to large, continuous forest patches. In these areas, the vegetation is continuous and less fragmented, creating a stable internal environment. This stability allows the forest canopy and root systems to develop fully, acting as a “sponge” that effectively intercepts rainfall and reduces soil erosion.^27^^,^^28^ In contrast, the lower water conservation capacity observed in the western region is largely due to its isolation and fragmentation. Without sufficient ecological corridors, these scattered patches cannot effectively exchange matter and energy with the core network. This isolation makes them more vulnerable to environmental stress, leading to a decline in their ability to retain water.^29^ Therefore, the “structure-function coupling” we observed essentially confirms that a complete and well-connected network is the structural foundation for sustaining regional hydrological regulation.

### The “KL strategy”: tailored optimization for different landscapes

The methodological value of this study lies in the “KL Strategy,” which offers different solutions for the distinct problems found in the eastern and western regions. Traditional uniform optimization methods often fail because they ignore local differences. In the eastern region, where the ecological foundation is already strong, our strategy focuses on making the network more robust. By adding backup connections between important nodes, we ensure that if one path is blocked, water conservation services can still be maintained through alternative routes. This “strong-to-strong” connection improves the overall efficiency of the network. On the other hand, the primary challenge in the western region is the lack of connections. Simply planting more trees in isolated patches is not enough if they remain cut off from the system. Our strategy uses “low-resistance pathways” to build bridges that reconnect these isolated patches to the main network. This approach transforms the landscape from a scattered state into a cohesive system, allowing the “trapped” ecological potential of western sources to be released and utilized. The significant improvement in robustness metrics confirms that this targeted approach effectively resolves structural bottlenecks while reinforcing the core backbone.^30^

### Implications for regional policy and management

To translate our topological findings into actionable governance, we propose a “Topology-Guided Spatial Planning Framework” that aligns our scientific insights with key regional policies in the Taihang Mountains. First, the critical nodes identified by our PageRank and Coreness analysis, such as Wuling Mountain and Zhangshiyan, act as the functional backbone of the entire network. We recommend that regional authorities prioritize these hubs for inclusion in the “Strict Ecological Conservation Redline.” By enforcing zero-development mandates in these specific areas, policy-makers can secure the core of the regional water supply system with minimal administrative cost. This ensures the long-term stability of the regional “sponge effect” by protecting the most influential nodes from degradation.^31^ Second, addressing the connectivity decline near the Jin-Ji-Lu-Yu urban cluster requires a pragmatic restoration approach. Instead of high-cost, continuous reforestation, we advocate for the restoration of newly identified optimal corridors through a “Stepping Stone” model. We propose strategically restoring small forest patches (1–2 *km*^2^) at topological bottlenecks. This strategy makes the addition of new linkages feasible within the constrained land-use budgets of the Beijing-Tianjin-Hebei Coordinated Development area, maximizing the efficiency of hydrological connectivity without creating severe land-use conflicts.^32^ Finally, regarding management implementation, we suggest moving away from a “one-size-fits-all” approach toward differentiated zoning. Newly proposed links that connect high-function hubs but currently face high resistance should be prioritized for Targeted Ecological Compensation funds. By focusing restoration efforts on these most efficient structural pathways, planners can optimize the spatial flow of water conservation services. This ensures that limited financial resources are directed toward the nodes and links that generate the highest functional impact, rather than being spread thinly across the landscape.^33^

### Limitations of the study

While this study provides a robust framework, we acknowledge several limitations that open avenues for future research. First, there are inherent limitations in the data precision and the mechanistic representation of the resistance surface. To ensure spatial consistency across the Taihang Mountains, all multi-source datasets were harmonized to a 200 m resolution under the WGS 1984 Albers projection. However, resampling coarser environmental variables (such as 1 km meteorological products) to a higher resolution does not enhance their inherent precision, which may introduce a degree of spatial smoothing and uncertainty in pixel-level water conservation assessments. Furthermore, our ecological resistance surface is primarily based on land-cover proxies. Future research should prioritize the integration of high-resolution soil hydraulic properties (e.g., saturated hydraulic conductivity) and field-based biological data to create more mechanistically robust models that accurately reflect actual hydrological and ecological flows. Second, our optimization strategy prioritizes ecological effectiveness. For practical implementation, a crucial next step would be to conduct a socio-economic cost-benefit analysis. This could involve using tools such as Marxan or Zonation to incorporate land acquisition costs, opportunity costs for agriculture, and stakeholder preferences, thereby identifying corridors that are not only ecologically optimal but also socially and economically feasible. Third, this study presents a static optimization for the year 2020. Future research should transition from static structural assessment to dynamic functional optimization. By employing landscape simulation models (e.g., LANDIS-II), we can project how the co-evolution of network structure and water conservation responds to future climate and land-use scenarios. A key priority is to optimize network configurations for resilience against extreme events, such as prolonged droughts or intense floods. Furthermore, moving toward a multi-objective approach that integrates water conservation with biodiversity and carbon sequestration will allow for the identification of functional synergies and the mitigation of negative trade-offs. This holistic perspective will ensure the long-term health of the landscape and provide a more robust framework for securing regional water resources in a changing environment.^6^

This study proposed and tested a structure-function coupling framework to advance the hydrological service-oriented optimization of ecological networks. A primary contribution of this research is the empirical confirmation that a strong, quantitative link exists between the topological structure of the forest-grassland network and its water conservation capacity. This finding provides a crucial mechanistic basis for conservation, suggesting that planning efforts can move beyond generic connectivity goals toward designing networks for specific, measurable functional outcomes.

Furthermore, we demonstrated that our KL strategy, tailored to the region’s landscape heterogeneity, is highly effective in enhancing ecosystem resilience. The targeted addition of critical corridors significantly improved the network’s structural robustness against disturbances. Methodologically, this result validates the efficacy of a spatially explicit, function-oriented approach over uniform conservation strategies.

In conclusion, this research offers both a deeper scientific understanding of the structure-function relationship in complex landscapes and a practical, transferable framework for evidence-based environmental management. While applied to water conservation in a mountain system, its principles hold strong potential for adaptation to other critical services and diverse landscapes. As such, this work contributes to the broader scientific effort of building more resilient and multifunctional landscapes in an era of accelerating environmental change.

## Resource availability

### Lead contact

Further information and requests for resources should be directed to and will be fulfilled by the lead contact, Teng Niu (niuteng21@stdu.edu.cn).

### Materials availability

This study did not generate new unique reagents.

### Data and code availability

• Data: All data reported in this paper will be shared by the lead contact upon reasonable request.
• Code: This paper does not report original code.
• All other items: Any additional information required to reanalyze the data reported in this paper is available from the lead contact upon request.

## Acknowledgments

This research is funded by the Hebei Provincial Social Science Development Research Project (grant no. 20220303176) and the Humanities and Social Sciences Project of 10.13039/501100003482Hebei Provincial Department of Education (grant no. SQ2024056). We also gratefully acknowledge the anonymous reviewers and editors for their constructive comments and suggestions that significantly improved this manuscript.

## Author contributions

Conceptualization, C.Z., J.Y., and T.N.; methodology, C.Z. and T.N.; software, H.S.; validation, Z.H., Y.W., and T.N.; investigation, J.Y.; data curation, Z.Z.; writing – original draft, C.Z.; writing – review and editing, T.N.; visualization, C.Z.; supervision, T.G.; project administration, Y.Z.

## Declaration of interests

The authors declare no competing interests.

## STAR★Methods

### Key resources table

**Table:** 

REAGENT or RESOURCE	SOURCE	IDENTIFIER
**Deposited Data**		

Land Use/Cover Data (LULC, 2000–2020)	Resource and Environmental Science and Data Center (RESDC)	https://www.resdc.cn/
Digital Elevation Model (DEM, 90 m)	Geospatial Data Cloud (SRTM)	http://www.gscloud.cn/
Meteorological Data (Precipitation, Temperature)	Cold and Arid Regions Scientific Data Center (CARS), CAS	http://data.tpdc.ac.cn/
Normalized Difference Vegetation Index (NDVI, MOD13Q1)	Index (NDVI, MOD13Q1)NASA/USGS (MODIS)	https://lpdaac.usgs.gov/
Soil Type Data (1:1,000,000)	Chinese Academy of Sciences (CAS)	http://www.soil.csdb.cn/
Road Network & Water System (Vector)	National Geomatics Center of China	https://www.ngcc.cn/

**Software and Algorithms**		

ArcGIS Desktop	Esri	v10.8
InVEST Model (Water Yield Module)	Natural Capital Project	v3.13.0
Python	Python Software Foundation	v3.8
NetworkX Library	Python Library	https://networkx.org/
Origin	OriginLab	

### Experimental model and study participant details

Not applicable, this study did not involve the use of experimental models or participants.

### Method details

#### Construction of ecological network

##### Extraction of ecological sources

Land that is ecologically important and sensitive is usually referred to as “ecological source land”.^9^ According to the source-sink theory in landscape ecology, ecological sources provide a variety of ecosystem services.^34^ This study focuses on the ecological space of forests and grasslands in the Taihang Mountains, selecting three land types— forested land, shrubland, and high-cover grassland—that typically contain water sources, as alternative ecological sources. Using national land-use data from 2000, 2005, 2010, 2015, and 2020, we first extracted forested land, shrubland, and high-cover grassland patches as potential alternative ecological sources.^35^^,^^36^ To ensure that selected sources are large enough to maintain internal ecosystem stability and serve as effective core habitats, a minimum area threshold was applied. Based on previous studies on landscape pattern and ecosystem services in the Taihang Mountains,^23^^,^^24^ which identified patches larger than 3–5 km^2^ as critical for regional ecological security and biodiversity, we adopted a conservative threshold of 4 km^2^.^37^ To evaluate the importance of each patch beyond its area, we calculated the ecological energy value (*E*_*i*_) of each alternative source using the entropy weight method to ensure objectivity. The calculation formula is as follows^26^:

(Equation 1) $$\begin{array}{l} E_{i}=\sum_{j=1}^{5}w_{j}\cdot x_{ij} \end{array}$$

Where *E*_*i*_ is the ecological energy value of source *i*;*w*_*j*_ represents the weights of the five selected indicators: patch area (0.3384), patch shape index (0.1051), average NDVI (0.0159), soil organic matter (0.2556), and biodiversity (0.2850); and *x*_*ij*_ is the standardized value of indicator *j*. Notably, the low weight assigned to NDVI (0.0159) results from the inherent logic of the EWM, which determines weight based on data variability. Since our ecological sources consist primarily of high-quality forests and grasslands with uniformly high vegetation cover, the minimal spatial variance of NDVI leads to a smaller objective weight compared to more heterogeneous factors such as biodiversity.

The distribution of ecological energy values among patches exhibits significant spatial heterogeneity. To further characterize this distribution, we conducted a statistical analysis (Table 2). The results show a skewness value of 16.42, indicating a robust right-skewed “long-tail” distribution. This statistical evidence confirms that the ecological energy follows both Poisson and power-law distributions. Based on these characteristics, we identified the top 50% of patches with the highest energy values as the core ecological sources. Notably, these top 50% patches, while only half in number, account for 92.4% of the total regional ecological energy (below table), ensuring that the selected sources represent the functional core of the study area.

**Table:** Statistical characteristics of ecological energy distribution

Statistic	Value	Description
Mean	88451.25	represents the average energy level of patches
Skewness	16.42	>0, indicating a significant right-skewed “long-tail” distribution
Kurtosis	338.56	reflects high concentration in the low-value range
Top 50% patches energy share	92.4%	validates the threshold for selecting core sources

#### Construction of an ecological network resistance ground

Ecological sources do not exist in isolation; they rely on the landscape matrix to facilitate the flow of energy and ecological processes. Following the identification of forest-grassland sources, the next critical step is to construct a resistance surface that quantifies the obstacles these ecological flows encounter. In national ecosystems, resistance factors create complex surfaces that reflect trends in energy flow and ecosystem service delivery^35^^,^^38^ To accurately simulate the movement of ecological flows in the Taihang Mountains, we selected six major resistance indicators: Land Use, Slope, NDVI, Road Network Density, Water Network Density, and Soil Organic Matter. These factors were chosen because they fundamentally determine the spatial heterogeneity of hydrological regulation and biological migration.^39^^,^^40^

The assignment of resistance values followed a standardized 1–9 scoring system, adhering to established methodologies.^25^^,^^26^^,^^41^ Specifically, lower resistance values (Level 1) were assigned to core habitats (e.g., forests, high-coverage grasslands) and areas with favorable environmental conditions (e.g., NDVI >0.85, Soil Organic Matter >6.82%), as these features facilitate the free movement of ecological flows. Conversely, higher resistance values (Level 9) were attributed to impermeable surfaces (e.g., urban land), steep terrains (Slope >18°), and areas with intense anthropogenic disturbance. Notably, the impact of linear features was differentiated: high water network density was treated as a facilitator of hydrological connectivity (low resistance), whereas high road density was identified as a significant barrier (high resistance). The detailed classification criteria and resistance assignments are presented in below table.^42^^,^^43^

**Table:** Evaluation system of ecological resistance factors

Evaluation factor	Classification criterion	Ecological resistance assignment
Land use	forest land, shrubland, high coverage grassland	1
	sparse woodland, other woodland, medium coverage grassland, rivers, canals, lakes, reservoirs, ponds, tidal flats, floodplains	3
	paddy field, dryland, low coverage grassland	5
	rural settlement, marshland, bare soil	7
	sandy land, desert, bare rocky land	8
	permanent glacier, urban land, other construction land, sandy land, saline-alkali land, ocean	9
Slope	0–3	1
	3–6	3
	6–9	5
	9–18	7
	>18	9
NDVI	0–0.19	9
	0.19–0.42	7
	0.42–0.66	5
	0.66–0.85	3
	>0.85	1
Road density	0–0.01	1
	0.01–0.03	3
	0.03–0.06	5
	0.06–0.09	7
	0.09–1.16	9
	impervious surface	9
Water density	0–0.16	9
	0.16–0.48	7
	0.48–0.87	5
	0.87–1.53	3
	1.53–3.57	1
Soil organic matter	0–0.51	9
	0.51–1.42	7
	1.42–3.1	5
	3.1–6.82	3
	>6.82	1

Based on the comprehensive resistance surface derived from these six factors, we employed the Minimum Cumulative Resistance (MCR) model to calculate the cost of movement from sources to any point in the landscape.^36^^,^^44^ This model serves as the basis for the subsequent extraction of ecological corridors. The calculation formula is:

(Equation 2) $$\begin{array}{l} V_{MCR}=f_{\min}\sum_{j=n}^{i=m}\left(D_{ij}R_{i}\right) \end{array}$$

In the equation, *V*_*MCR*_ is the value of the MCR, *f*_*min*_ is the minimum value of the cumulative resistance of the land unit, *D*_*ij*_ is the spatial distance from the ecological source to *j* the land unit *i* and *R*_*i*_ is the resistance coefficient of the land unit *i* to the movement process.

#### Construction of complex networks

The construction of the ecological network is fundamentally grounded in the theory of landscape energy flow, where ecological sources facilitate ecological processes while the resistance surface inhibits them. The “minimum consumption distance” represents the cumulative energy cost required for organisms or matter to traverse the resistance surface from one source to another. Ecological corridors are, in essence, the spatial manifestation of these least-cost paths, selected to maximize ecological benefits by connecting sources through optimal routes.^10^ To spatially delineate these pathways, we employed the Ecological Network Builder (Graphab) based on the Cost-Path Model to extract potential corridors linking the forest-grassland sources identified in Section 2.3.1.

The interconnection of these corridors and sources forms a spatial system that exhibits typical characteristics of complex networks, such as small-world properties.^6^ However, treating this system as a simple unweighted (binary) graph would oversimplify the reality, as ecological sources differ significantly in quality and corridors vary in resistance cost. To accurately capture this functional heterogeneity, we upgraded the binary graph into a weighted complex network.^45^^,^^46^ In this empowered model, the interaction intensity (edge weight) between sources is quantified using the Gravity Model.^47^ This model posits that the interaction strength (*w*_*ij*_) between two sources is proportional to their ecological energy and inversely proportional to the cumulative resistance of the corridor connecting them:

(Equation 3) $$\begin{array}{l} w_{ij}=\frac{I_{i}I_{j}}{L_{ij}^{2}} \end{array}$$

In the equation, *w*_*ij*_ is the edge right connecting ecological source *i* and ecological source *j*, *I*_*i*_ and *I*_*j*_ are the ecological energy of ecological sources *i* and *j*, and *L*_*ij*_ is the cumulative resistance value of the ecological corridor connecting node *i* and node *j*.

The weight of an ecological source is defined as the sum of the weights of all ecological corridors connected to it, and is a natural extension of the degree of nodes in an unweighted network,^48^ with the node weight formula expressed as:

(Equation 4) $$\begin{array}{l} S_{i}=\sum_{j=1}^{N}w_{ij} \end{array}$$

In the equation, *S*_*i*_ is the weight of ecological source *i*, and *N* is the number of neighboring sources of ecological source *i*.

#### Topological structure of ecological spatial network

The topological structure of ecological spatial networks is crucial for maintaining regional ecological security, directly influencing the continuity and stability of ecological processes. Unlike traditional geographic networks, ecological sources and corridors support multiple ecological functions, including material, energy, and biological flows.^49^^,^^50^ This paper applies complex network theory to ecological spatial research by analysing six topological indicators: degree, node weight, unit weight, coreness, PageRank value and betweenness.^51^^,^^52^^,^^53^^,^^54^ Degree reflects the direct connectivity of nodes, while node weight and unit weight quantify the ecological attributes of nodes and the resistance characteristics of edges. Coreness reveals the network’s hierarchical structure, while PageRank and betweenness represent the nodes’ global influence and control. This combination of multidimensional indicators provides a comprehensive capture of the topological nature of the ecospatial network, revealing underlying patterns of ecological source connectivity and offering a quantitative basis for optimising network structure.

#### Water source conservation services and related characteristics

##### Water-sourcing ecosystem services

Water conservation serves as the core indicator for measuring the functionality and ecological benefits of the forest-grassland network. At the national scale, the forest-grassland ecological space in the Taihang Mountains functions as a critical strategic barrier for maintaining regional water security. To scientifically quantify this capacity and validate the network’s effectiveness, a robust assessment method is required. The Water Balance Equation is the primary method for assessing the water conservation capacity of ecosystems. Based on the principle of mass conservation, it states that the amount of incoming water is equal to the sum of outgoing water and the water stored within the system over a specific period and in a specific area.^34^^,^^55^ The basic equation of the water balance method is as follows:

(Equation 5) $$\begin{array}{l} Q_{WCC}=\sum_{i=1}^{j}\left(P_{i}-R_{i}-ET_{i}\right)\times A_{i} \end{array}$$

In the formula: *Q*_*WCC*_ represents the water containment capacity (m^3^), *P*_*i*_ is precipitation (mm), *R*_*i*_ is stormwater run off (mm), *ET*_*i*_ is evapotranspiration (mm), *A*_*i*_ is the area of ecosystem type *i* (m^2^), and *j* is the number of ecosystem types.

When evaluating regional ecosystem water conservation capacity based on grid cells, without considering the area of the ecosystem, the result obtained is the ecosystem water conservation volume (mm), as expressed by the formula:

(Equation 6) $$\begin{array}{l} Q_{WCC}=P-R-ET \end{array}$$

The main indicator factors required for the calculation of the water sources in the calculation are shown later in discussion:

The actual evapotranspiration is calculated using the Zhang model based on Budyko’s assumptions of water-heat balance, and the main formulas for its calculation are shown below^56^^,^^57^^,^^58^

(Equation 7) $$\begin{array}{l} ET=P\times \frac{1+w\times \frac{PET}{P}}{1+w\times \frac{PET}{P}+\frac{P}{PET}} \end{array}$$

In the equation, *ET* is the actual evapotranspiration; *P* is the precipitation; *w* is the water use coefficient for a particular land use type; and *PET* is the potential evapotranspiration, which is calculated as follows:

(Equation 8) $$\begin{array}{l} PET=0.162\times \frac{SR}{58.5}\left(DT+17.8\right) \end{array}$$

In the equation, *SR* is the monthly average total solar radiation for each month; *DT* is the monthly average temperature for each month.^59^

#### Quantifying structure-function coupling via statistical correlation

At the national scale, the forest-grassland ecological space, formed by ecological source land in the ecological network, provides typical water-sourcing and nutrient-supporting services. These services are exchanged and transmitted through ecological corridors in the spatial network. To characterize the coupling between network structure and hydrological function, the Pearson correlation coefficient was employed as a quantitative evidence to reflect the synergy between indicators. This linkage demonstrates how the network’s topological configuration (structure) and water services (function) are functionally coupled, where the correlation strength indicates the degree of functional response to structural properties.This coefficient was then used to evaluate the correlation between ecological network topological indicators and water sourcing and nutrient supporting services over the period 2000–2020. A unit-by-unit spatial analysis was conducted to calculate the correlation between stabilised ecological source water and nutrient services, and topological indicators, over twenty consecutive years. The Pearson correlation coefficient is calculated as follows:

(Equation 9) $$\begin{array}{l} R=\frac{\sum \left(x-\bar{x}\right)\left(y-\bar{y}\right)}{\sqrt{\sum {\left(x-\bar{x}\right)}^{2}\sum {\left(y-\bar{y}\right)}^{2}}} \end{array}$$

In the equation, *R* is the correlation coefficient.When |R|>R_0.05_, which *p* < 0.05, the original hypothesis is rejected and the correlation result is significant, and When |R|>R_0.01_, which *p* < 0.01, the original hypothesis is rejected and the correlation result is highly significant.

#### The Keystone-Linkage (KL) optimization strategy and robustness assessment

##### The Keystone-Linkage (KL) optimization strategy

To guide the network optimization, we developed the KL strategy. This systematic approach is founded on the ecological principle of identifying ‘keystone’ sources—the most critical nodes for network function—and strategically enhancing the ‘linkages’ (corridors) between them. The KL strategy is differentiated, applying distinct corridor addition tactics to the network’s core and peripheral zones to maximize functional gains in water conservation.This is achieved by adding new ecological corridors based on the “Topology-Function Coupling” theory to improve the ecological network structure of the Taihang Mountain region.^30^ The strategy builds on previous analysis of the correlation between ecological sources, resistance surfaces, topological features, and water conservation, with the core objective of “strengthening ecological connectivity to enhance water conservation efficiency”. It offers a practical approach to optimising the regional ecological network through the following process: screening Pearson’s correlation results between high-priority ecological networks based on topological indicators and water conservation functions to identify significant relationships (below figure). Four indicators with a significant impact on water conservation are selected: highly significant positive correlations in degree (R > 0.6), node weight (R > 0.6), PageRank value (R > 0.6), and significant positive correlation in unit weight (R = 0.1–0.4). A comprehensive attribute evaluation model is then constructed^31^:

(Equation 10) $$\begin{array}{l} S_{i}=\sum_{k=1}^{4}w_{k}\cdot X_{ik} \end{array}$$

In the equation, *S*_*i*_ is the comprehensive attribute value of the *i* ecological source, *w*_*k*_ is the weight of each index (degree 27%, node weight 30%, PageRank 31%, unit weight 12%, based on the absolute value of the correlation coefficient normalized), and X_ik_ is the standardized value of the index. The model calculates a comprehensive attribute value for each source to identify high-priority nodes for optimization (below figure). To apply the Pareto principle, which suggests that roughly 80% of effects come from 20% of the causes, we focused on identifying the ‘vital few’ sources that contribute most significantly to the network’s function. We ranked all sources by their comprehensive attribute value and selected the top 20% as high-priority optimization targets. This approach ensures that conservation efforts are directed toward the most influential nodes—those with the highest connectivity and ecological quality—thereby maximizing the efficiency and impact of the optimization strategy. These sources represent the critical backbone of the network, and strengthening their connections is expected to yield the greatest functional return.

**Figure:**
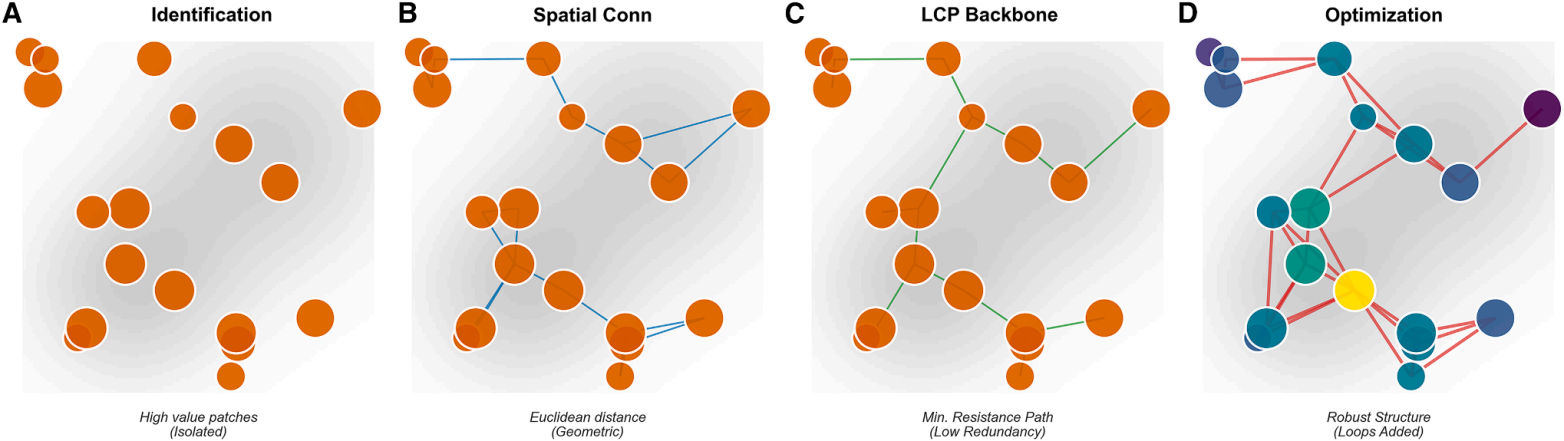
Evolutionary process of ecological network construction and KL-based optimization (A–D) Panels illustrate the transition from source identification and geometric connectivity to the MCR-based backbone and final optimized robust structure. Node sizes and colors reflect patch area and hierarchical importance, respectively; gray shading denotes landscape resistance.

**Figure:**
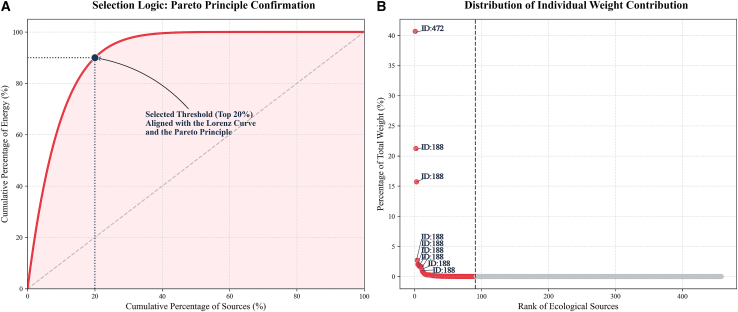
Rationale and distribution analysis for the selection of keystone ecological sources (A) Selection rationale: validation of the Pareto Principle through the Lorenz Curve. (B) Rank-size distribution of individual source weight contributions.

#### Robustness assessment

To quantitatively evaluate the effectiveness of this optimization, we assessed the network’s robustness before and after optimization (using 2020 as the baseline). Robustness is defined as the network’s ability to maintain structural and functional stability under disturbances.^60^ We simulated both malicious attacks (targeting high-degree nodes) and random attacks using the following metrics:

Connectivity Robustness (*R*): This metric measures the ability of the remaining nodes to stay connected after destruction:

(Equation 11) $$\begin{array}{l} R=\frac{C}{N-N_{r}}j \end{array}$$

In the formula, *R* is the connectivity robustness, *C* is the number of nodes in the connected sub-network after destruction, *N* is the total number of nodes in the network, and *N*_*r*_ is the number of removed nodes. Through this formula, the network can be quantitatively assessed under random attacks (such as random deletion of nodes), malicious attacks (priority deletion of important nodes, such as high connectivity ecological source), and connectivity decay law, to determine the network’s resistance to node damage “resilience threshold”.

Recovery Robustness (*D* and *E*): Evaluates the self-repair potential of nodes (*D*) and edges (*E*) after damage:

(Equation 12) $$\begin{array}{l} D=\frac{N_{r}}{N-{N_{r}}^{\prime }}\;and\;E=\frac{E_{r}}{E-{E_{r}}^{\prime }} \end{array}$$

Where ${N_{r}}^{\prime }$ and ${E_{r}}^{\prime }$ represent the number of nodes and edges that can be recovered after the removal of *N*_*r*_ nodes and *E*_*r*_ edges, respectively.

By quantifying the enhancement in these metrics, we validated whether the optimized “Core Reinforcement-Edge Penetration” pattern effectively supports regional water security.

### Quantification and statistical analysis

Statistical analyses and data visualization were performed using Python (v3.8) and Origin. The relationship between topological metrics (e.g., Degree, PageRank) and water conservation capacity was evaluated using Pearson correlation analysis. In this study, n represents the number of ecological source nodes, with an exact value of *n* = 452 (based on the 2020 baseline network). Statistical significance was defined as *p* < 0.05, and highly significant results were defined as *p* < 0.01. No specific method was used to predetermine sample size, as it was determined by the identified ecological network components. Detailed statistical results, including exact *p* values and correlation coefficients (r), can be found in the Results section, relevant Figure Legends, and Tables.
